# An Open-Source Restraint System for Magnetic Resonance Imaging in Awake Rats

**DOI:** 10.1523/ENEURO.0390-25.2026

**Published:** 2026-02-25

**Authors:** Richard Quansah Amissah, Mahmoud Khaled Hanafy, Hakan Kayir, Peter Zeman, Kyle Gilbert, Miranda Bellyou, Amr Eed, Colette E. Mahr, Ashley L. Schormans, Brian L. Allman, Jibran Y. Khokhar

**Affiliations:** ^1^Department of Anatomy and Cell Biology, Schulich School of Medicine and Dentistry, Western University, London, Ontario N6A 3K7, Canada; ^2^Center for Functional and Metabolic Mapping, Robarts Research Institute, Western University, London, Ontario N6A 3K7, Canada

**Keywords:** functional, head-fixed, MRI, preclinical, rodent

## Abstract

Magnetic resonance imaging (MRI) is a critical tool for translational neuroscience, but preclinical studies frequently rely on anesthesia, which alters neural activity and limits comparison with human studies. Awake rodent functional MRI (fMRI) enables investigation of brain function under physiologically relevant conditions; however, its implementation is constrained by the need for anesthesia during restraint setup. We developed and evaluated a restraint system and habituation protocol for awake rat fMRI. Ten rats were studied: an awake group and an anesthetized group (three males and two females per group). The protocol included head post implantation and an 11 d habituation period. T2-weighted anatomical and functional scans were acquired. Head motion and functional connectivity were analyzed using the RABIES pipeline and compared between groups. The modular 3D-printed restraint system developed can be assembled in under 5 min; eliminates the need for anesthesia, ear bars, and bite bars; and supports several behavioral paradigms. High-quality anatomical and functional images were obtained for awake rats. Anesthetized rats exhibited significantly lower translation, rotation, and framewise displacement. Functional connectivity differed between awake and anesthetized rats, with some region pairs showing higher (e.g., left–right primary somatosensory cortex and hypothalamus–insula) and lower (e.g., cingulate–prelimbic cortex and retrosplenial–motor cortex) correlations in awake rats. However, these differences did not survive network-based statistics correction. This work presents a scalable, reproducible, and animal-friendly platform for awake rat fMRI that enables high-quality, behaviorally enriched imaging without anesthesia, while highlighting the effects of anesthesia on functional connectivity.

## Significance Statement

Most rodent fMRI studies, including awake studies, rely on anesthesia, which profoundly alters brain activity and limits the interpretation of the data. This study presents a novel restraint system that enables high-quality fMRI in fully awake rats, eliminating the need for anesthesia, ear bars, and bite bars. By reducing motion, this simple restraint system allows for investigation of neural activity and connectivity without confounds from sedation or anesthesia. Its open-source, modular design supports behavioral tasks and broad accessibility, making it a valuable tool for neuroscience research seeking to bridge the gap between preclinical imaging and real-world brain function.

## Introduction

Functional magnetic resonance imaging (fMRI) has become a valuable tool in translational neuroscience, offering noninvasive, whole-brain mapping of neural activity via blood-oxygen-level-dependent (BOLD) signal (Kwong et al., 1992). Unlike other methods such as electrophysiology, mini-scope imaging, and fiber photometry, fMRI provides superior spatial resolution and brain-wide coverage, enabling the investigation of distributed networks during rest and behavior, in addition to being akin to signals captured in human populations (Glover, 2011; Rizzolatti et al., 2018). Most rodent fMRI studies, however, rely on anesthesia or sedation to reduce motion and stress during scanning. While effective for immobilization, anesthesia alters neurovascular coupling, suppresses neural activity, and disrupts functional connectivity, thereby limiting interpretability of functional imaging data (Sloan, 1998; Martin et al., 2006; Paasonen et al., 2018).

Awake rodent fMRI avoids these confounds, allowing researchers to examine brain function under more naturalistic conditions (Ferris, 2022). Despite its advantage, widespread adoption has been hindered by technical barriers, including procedural complexity, lack of standardized protocols, and reliance on custom hardware, which is often required to achieve adequate signal quality and head stabilization. Existing restraint systems for awake imaging are generally classified as invasive or noninvasive, each with trade-offs between motion control and animal welfare. While several systems have been developed for mice (Harris et al., 2015; Yoshida et al., 2016; Madularu et al., 2017; Chen et al., 2020; Fadel et al., 2022; Gutierrez-Barragan et al., 2022; Mikkelsen et al., 2022; Laxer et al., 2025), many require brief anesthesia during habituation and/or scanning. Restraint-based systems for rats remain relatively underdeveloped due to their larger size and greater strength and often still depend on initial anesthesia (King et al., 2005; Febo, 2011; Upadhyay et al., 2011; Chang et al., 2016; Stenroos et al., 2018; Ma et al., 2020; Russo et al., 2021; Wallin et al., 2021).

In this study, we present a detailed, reproducible protocol for conducting awake fMRI in rats using a custom-designed, modular restraint system. Our approach combines skull-screw-less head post implantation with a structured 11 d habituation regimen to minimize motion without the use of anesthesia. The system is compatible with behavioral tasks and supports stable imaging suitable for longitudinal studies. By addressing both technical and welfare challenges, our method enhances the reproducibility and translational relevance of rodent fMRI research.

## Materials and Methods

### Animals

All experimental procedures were approved by Western University Animal Use Committee (Approval Number: 2025-001) and conducted in accordance with both provincial (Ontario Ministry of Agriculture, Food and Agribusiness; OMAFA) and federal (Canadian Council on Animal Care; CCAC) guidelines. Ten 6-month-old rats divided into two groups (anesthetized and awake groups) were used for this study. The awake group comprised five rats (male: one Sprague Dawley and two Long–Evans; female: two Long–Evans), while the anesthetized group comprised five wild-type cell adhesion molecule 2 (cadm2) long deletion rats (three males and two females). Animals were housed in a controlled environment maintained at 21 ± 2°C, with 30–40% relative humidity and a 12 h light/dark cycle (lights on at 07:00). Standard rodent chow (14% protein, Envigo) and water were available *ad libitum*. Rats received banana-flavored sugar pellets (Bio-Serv, 45 mg, banana flavor) as a reward after each habituation session.

### Restraint system design

**Figure 1.**
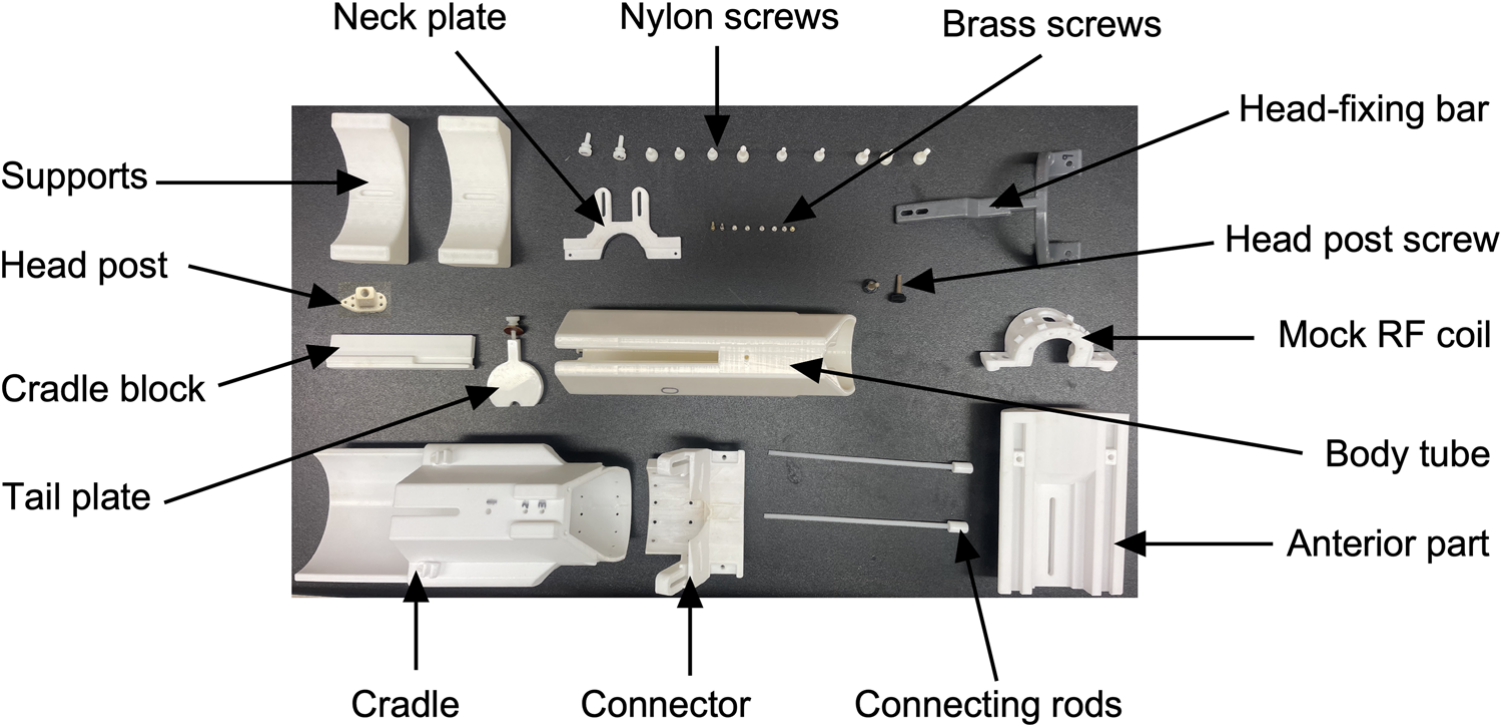
Components of the restraint system. The restraint system consists of 14 components: neck plate, nylon screws, brass screws, head-fixing bar, head post screw, mock radio frequency (RF) coil, body tube, anterior part, connecting rods, connector, cradle, tail plate, cradle block, and supports. The implanted head post used for head fixation is also shown. Except for the brass screws, all components were 3D printed in-house.

The entire restraint system (Fig. 1) was 3D printed. The computer-aided design (CAD) file has been included (https://doi.org/10.17605/OSF.IO/JQ6KS). With the exception of the head-fixing bar, which was printed using Tough 2000 Resin (FormLabs), all other components were printed with Tough Polylactic Acid (PLA; Shop3D). The head post and head post screw were printed using polyetheretherketone (PEEK) filament, while the plastic screws were printed from nylon filament (Shop3D). The head-fixing bar was printed over 2 d on a Form 3+ printer (FormLabs). The remaining PLA and polycarbonate components were printed on a RAISE3D Pro2 Plus printer (RAISE3D). After printing, the head-fixing bar was washed and cured. Brass screws were used to attach the connector to the cradle of the restraint system. The actual radiofrequency (RF) coil contains eight copper loops (Zanini et al., 2023). Postprocessing of all parts included drilling and sanding. The entire restraint system was printed over the course of 1 week using the printers’ fastest settings. The total cost of the system components was approximately CAD $350.

**Figure 2.**
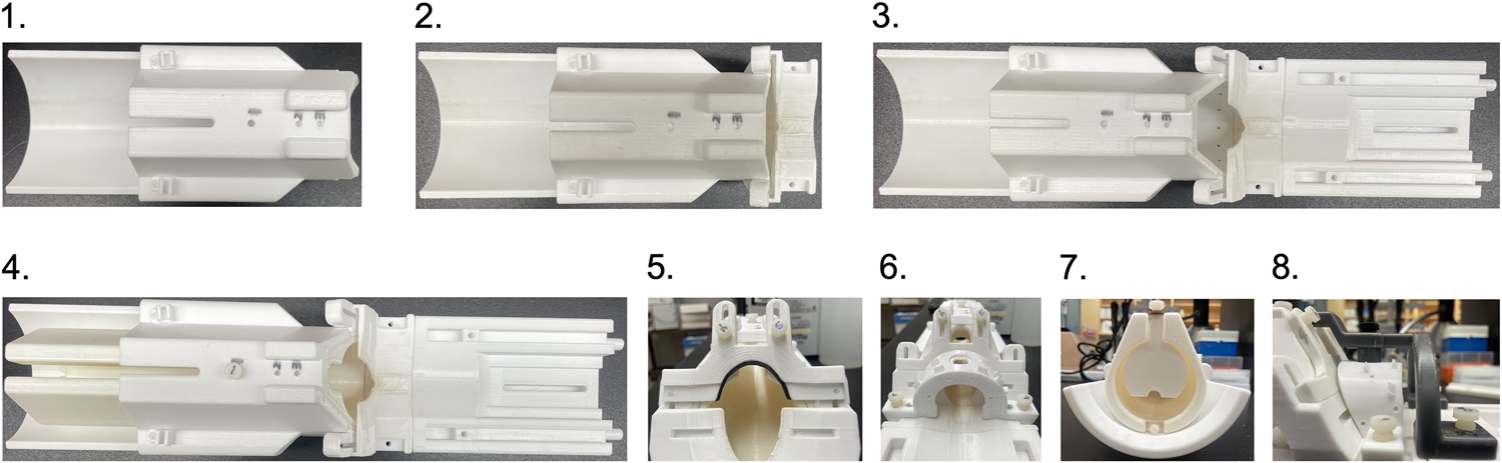
Assembly of the restraint system. Step-by-step images illustrating the simple, 8-step procedure for assembling the restraint system.

Steps to assemble the restraint system (Fig. 2)

1. Place the cradle on a stable surface.
2. Attach the connector to the cradle using the brass screws.
3. Attach the anterior part of the restraint system to the cradle and connector using the connecting rods.
4. Insert the body tube into the cradle and secure it with a nylon screw.
5. Place the neck plate into the cavity between the cradle and connector, and fasten it with nylon screws.
6. Position the mock RF coil on the anterior part of the connector and attach it with nylon screws.
7. Place the tail plate in the groove on the body tube and tighten its screw.
8. Fix the head-fixing bar to the anterior part of the restraint system. Ensure that the head post receptacle is aligned directly above the hole in the mock RF coil.
9. Secure the head-fixing bar to the cradle with nylon screws.

### Stereotaxic surgery

The rat was first weighed to calculate the appropriate dose of Metacam (meloxicam oral suspension, Boehringer Ingelheim Vetmedica) and Baytril (enrofloxacin, Elanco US) for administration during surgery. Anesthesia was induced with 5% isoflurane and 2% oxygen until the desired depth of anesthesia was reached. The rat was then injected with meloxicam (5 mg/kg) and Baytril (5 mg/kg). Next, the rat's head was shaved to expose the scalp. The rat was positioned on the stereotaxic apparatus, with a prewarmed heating pad used to maintain body temperature. A rectal probe was inserted to monitor body temperature continuously. Ear bars and a bite bar were used to stabilize the head. Eye gel was applied to prevent corneal drying. The shaved skin was disinfected sequentially with soap, isopropyl, and chlorhexidine to maintain a sterile field.

The isoflurane concentration was then reduced to 2%. A toe pinch was performed to confirm the absence of reflexes and ensure a sufficient depth of anesthesia. A midline scalp incision was made from the anterior to the posterior aspect of the head to expose the skull. Underlying tissues were removed, and the skull was cleaned to clearly expose the bregma and lambda. Etchings were made on the skull to enhance bone cement adhesion. The head post, stored in isopropyl until needed, was mounted onto the vertical arm of the stereotaxic apparatus and positioned over the lambda. Care was taken to avoid tilting the head post to ensure proper orientation during MRI scanning. A thin layer of All-Bond Universal (BISCO) was applied to the skull to improve bone cement adhesion and cured with UV light. Bone cement (Core-Flo DC Lite, BISCO) was then applied. The head post was lowered onto the skull so that its lower portion was embedded within the cement. Later, the thickened, yet still fluid, bone cement was smoothed using a spatula to eliminate sharp edges that could cause irritation after hardening and then cured.

Once the cement had hardened, the head post was detached from the stereotaxic arm. The scalp was repositioned over the bone cement, without requiring sutures. The area was cleaned of residual blood. Finally, 3 ml of saline was administered subcutaneously. Isoflurane was gradually turned off, while oxygen was maintained until the rat regained consciousness. The ear and bite bars were quickly removed at the first signs of regained consciousness. The rat was transferred to a recovery cage until it was fully mobile and then placed in a clean cage. Postoperative care included administration of Metacam and Baytril for 3 d. The rat was allowed a 10 d recovery period before restraint system habituation began.

### Statistics analyses

Head motion parameters were compared between anesthetized and awake rats using independent-samples *t* tests conducted in IBM Statistical Package for Social Sciences (SPSS) software version 23. Data are presented as mean ± standard deviation. Statistical significance was set at *p* < 0.05 for all comparisons.

We assessed group-level functional connectivity differences using Network-Based Statistics (NBS) in MATLAB. Atlas-based Pearson’s correlation matrices were generated for each subject and entered into mass univariate two-tailed *t* tests. A primary threshold of *t* = 2.05 defined suprathreshold connections, which were grouped into connected subnetworks. Cluster significance was evaluated with 10,000 permutations, controlling family-wise error at *p* < 0.05 by comparing observed cluster sizes to permutation maxima. Heat maps were plotted using a custom MATLAB script.

### Steps for habituation to restraint, head fixing, and scanner noise

**Figure 3.**
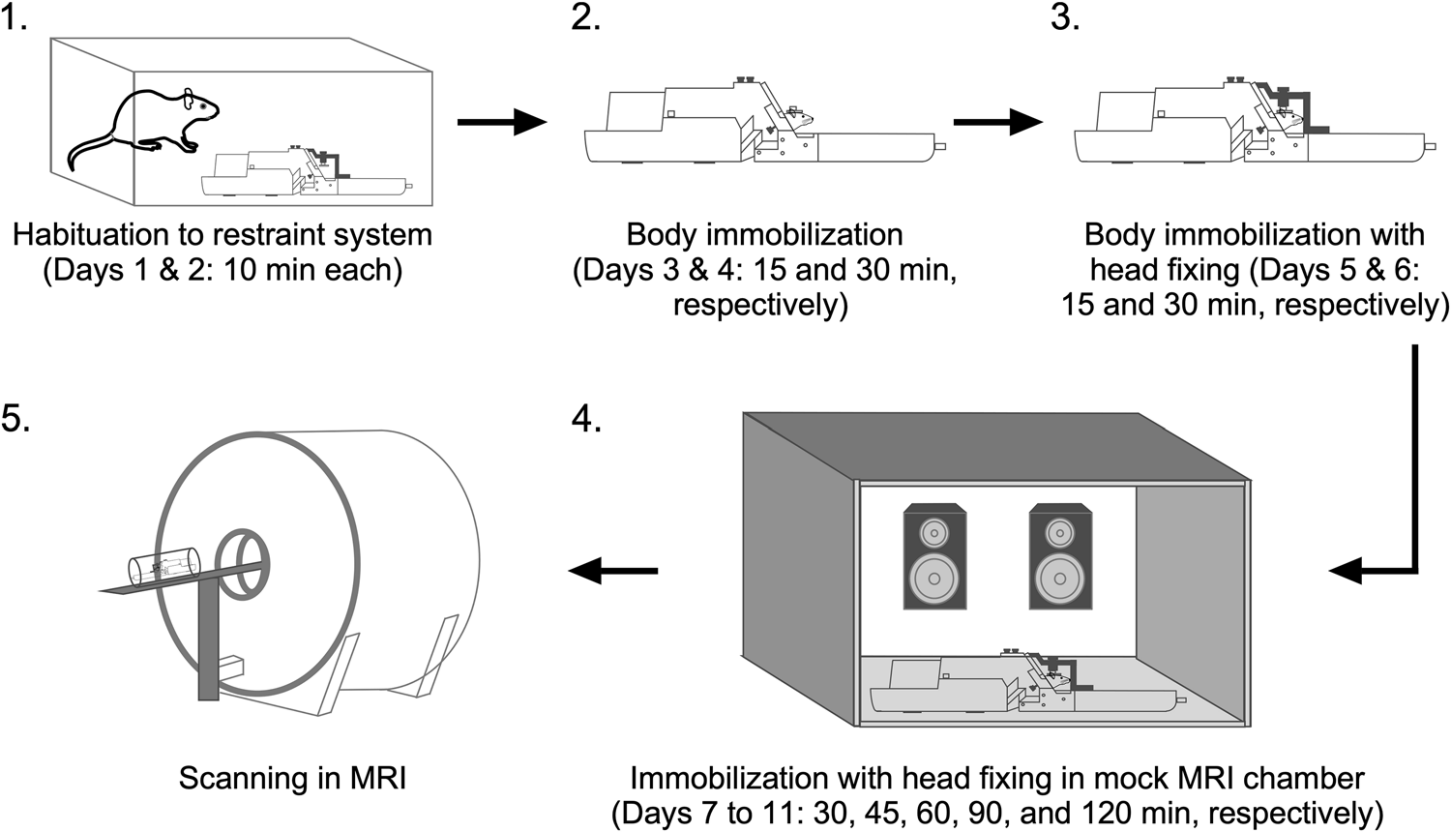
Visual flow diagram summarizing the step-by-step procedure for habituating a rat to the restraint system using the habituation protocol.

This protocol outlines a step-by-step method to habituate rats to restraint, head fixation, and scanner noise for awake fMRI studies (Fig. 3). It is designed to minimize movement while preserving physiologically relevant brain states. The entire process spans ∼11 d.

Before beginning the restraint protocol, habituate the rat to handling, head-holding by hand, and receiving a reward after restraint and head-fixing. Gradually increase the duration of the head-holding until you can comfortably hold the rat's head for ∼1 min.

**Figure 4.**
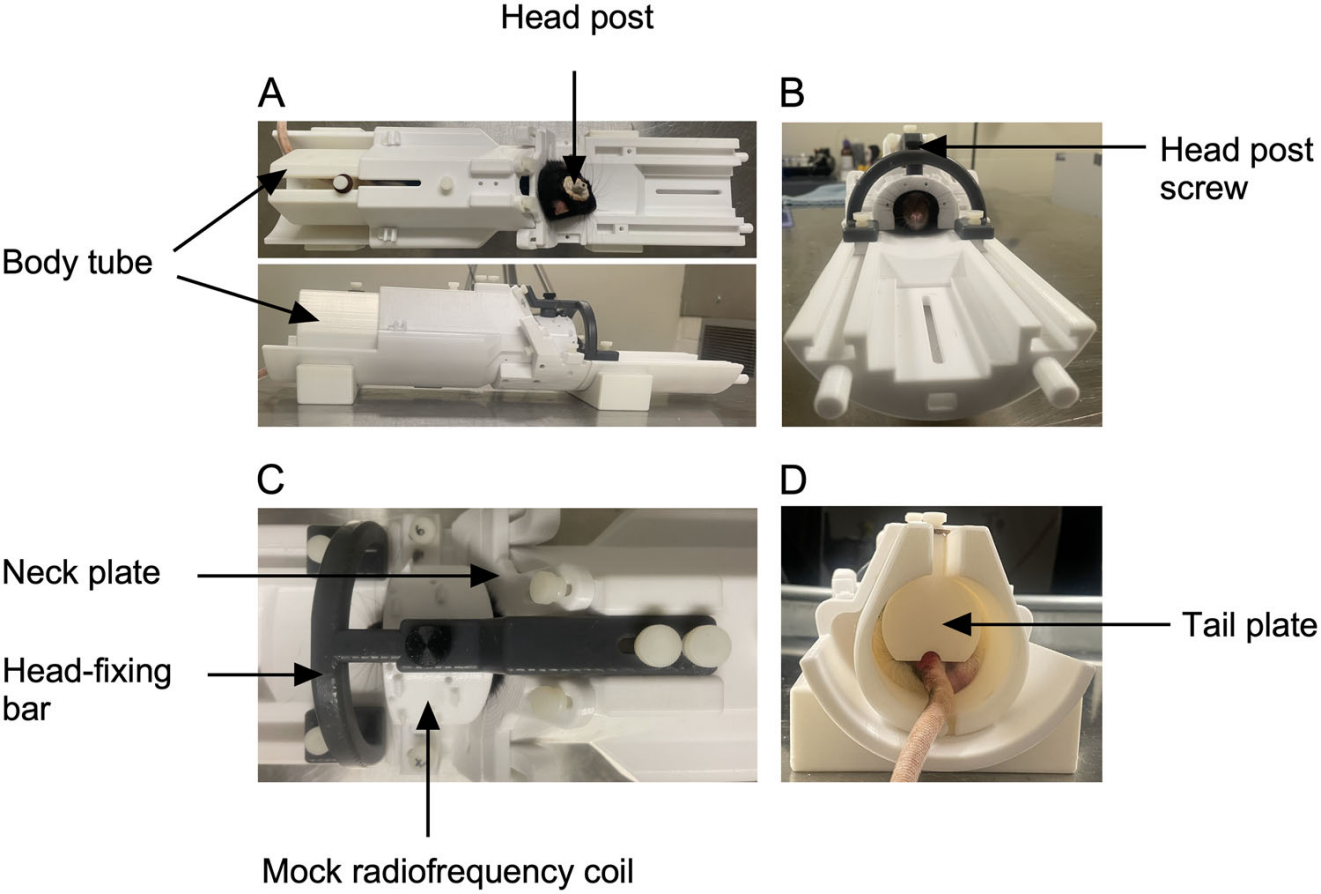
Head fixation of a rat in the restraint system (multiple views). ***A***, Top panel, Rat positioned in the restraint system with the neck plate secured. Bottom panel, Side view of the system after placement of the head-fixing bar, which is attached to the rat via the implanted head post and secured to the restraint system. ***B***, Front view showing the head-fixing bar secured to both the rat and the restraint system. ***C***, Top view showing details of the nylon screws and head post screw used to firmly immobilize the rat's head. ***D***, Rear view illustrating the tail plate and positioning of the rat's tail during head fixation and scanning.

1. Habituation to the restraint system (Days 1 and 2; Fig. 3, Step 1): Place the restraint system in an open field box and allow the rat to explore it for 10 min each day.
2. Body immobilization (Days 3 and 4; Fig. 3, Step 2): Immobilize the rat's body using the body tube, leaving the head unrestrained.
  i. Place the body tube halfway on a regular napkin, with the rear of the tube resting on the napkin.
  ii. Position the rat on the napkin with its head facing the rear of the body tube.
  iii. Wrap the napkin around the rear of the tube and the rear of the rat to encourage it to move forward through.
  iv. Once the rat enters the tube, lift the tube and gently insert it into the cradle.
  v. Raise the neck plate to encourage the rat to extend its head through the front opening of the cradle. If needed, use the tail plate to gently push the rat forward.
  vi. Once the rat positions its head under the neck plate, gently lower the neckplate and secure it with screws to prevent backward movement. When lowered, the neck plate engages at the level of the shoulder girdle/thoracic inlet. The plate and connector form an adjustable circular opening that fits around the neck without compressing the cervical airway, ensuring unobstructed breathing during restraint.
  vii. Insert the tail plate into its designated groove to prevent backward movement, and secure it tightly. Ensure the rat's tail passes through the tail plate grove to avoid injury.
  viii. Keep the rat in this position for 15 min on day 3 and 30 min on Day 4.
3. Body immobilization with head fixing (Days 5 and 6; Fig. 3, Step 3): Immobilize the rat as previously described and head-fix it.
  i. With the rat's head protruding from the neck plate, place the mock RF coil over the head so that the head post extends through the hole in the coil. Secure the coil to the restraint system.
  ii. Position the head-fixing bar so that its designated slot is directly above the head post.
  iii. Secure the head post to the head-fixing bar to prevent both vertical and horizontal head movement.
  iv. Attach the front and rear parts of the head-fixing bar to the restraint system to secure it (Fig. 4).
  v. Keep the rat immobilized and head-fixed for 15 min on day 5 and 30 min on Day 6.
4. Immobilization with head-fixing in mock MRI chamber (Days 7–11; Fig. 3, Step 4): After immobilization and head fixation, place the rat with the restraint system inside the mock MRI chamber and expose it to recorded MRI (T2-weighted anatomical and GE-EPI acquisition) sounds.
  i. Using a speaker capable of producing sounds up to 120 dB sound pressure level (SPL; the peak intensity of sounds produced by the MRI system used during both T2-weighted anatomical and GE-EPI sequences), play the recorded MRI sounds–starting at a lower intensity on Day 7 and gradually increasing the intensity each day, reaching full intensity by Day 11.
  ii. From Days 7 to 11, immobilize the rat with head-fixing in the mock MRI chamber for progressively longer durations: 30, 45, 60, 90, and 120 min, respectively. No overt behavioral differences, such as head movement, struggling, or agitation, were observed during exposure to MRI sounds generated during either T2-weighted anatomical scans or GE-EPI sequence.

After completing the 120 min restraint, the rat can be placed in the actual MRI scanner (Fig. 3, Step 5). However, repeating the 2 h restraint session several more times is recommended to further habituate the rat and reduce stress during actual scanning. Additionally, providing a reward after each restraint and/or head-fixing session is encouraged to create a positive association with the experience.

By the final day of habituation, rats were introduced into the restraint and head-fixation setup without resistance and exhibited minimal or no tail flicks, body twisting, or escape attempts. They also produced fewer fecal pellets during this session and consistently consumed the rewards offered after each session. These behaviors were used as qualitative criteria to determine completion of habituation. Across all animals exposed to the protocol, 100% (*n* = 5) successfully completed the full habituation protocol.

### Mock MRI chamber

The mock MRI chamber used in this study was designed as a double-walled sound booth, consisting of two Med Associates sound-attenuating cabinets of different sizes, with the smaller, internal chamber (ENV-022MD; Standard MDF cubicle) placed inside the larger one (ENV-017; Extra-large MDF cubicle). The interior walls of the internal chamber were lined with soundproofing foam sheets (2″ pyramidal shape; Foam Factory) to limit sound reflections and to minimize sound escaping from the small portals in the mock MRI chamber used for cable passage. Inside the chamber, a speaker capable of producing sound levels up to 120 dB SPL was installed. The speaker was connected to an external laptop used to play the recorded MRI sound file. The MRI sounds were recorded from a 9.4 T Bruker MRI scanner and included noises produced during shimming, T2-weighted image acquisition, and fMRI acquisition. The total duration of the recorded sounds was 2 h. Depending on the stage of habituation, the appropriate segment of the recording was played.

### MRI experiments

Imaging was performed at the Center for Functional and Metabolic Mapping, located within the Robarts Research Institute at Western University. Scans were obtained using a Bruker 9.4T, 31 cm horizontal-bore magnet (Varian/Agilent) equipped with a 6 cm Magnex HD gradient insert and a Bruker BioSpec Avance NEO console running ParaVision 360 v3.3 (Bruker BioSpin).

On the day of scanning, as described previously (Frie et al., 2024), rats in the anesthetized group were anesthetized in an induction chamber with 4–5% isoflurane in oxygen at a flow rate of 1–1.5 L/min. After induction, anesthesia was maintained with 1.5–2.5% isoflurane in oxygen (1–1.5 L/min) via a custom-built nose cone, and dexmedetomidine (0.018 mg/kg, intraperitoneal) was administered. Once positioned in the scanner, dexmedetomidine was continuously infused at 0.018 mg/kg/h for the duration of the scan. Following initiation of dexmedetomidine infusion, isoflurane was gradually reduced from 2.0–2.5 to 0.8–1.0% over a 15 min period while maintaining an oxygen flow rate of 1–1.5 L/min to ensure stable physiology (respiration rate, 74 ± 5.2 breaths per minute; heart rate, 364 ± 23 beats per minute). Rectal temperature was maintained at 37.0 ± 0.5°C using an air heater. As in previous studies, EPI acquisitions were initiated once optimal physiological conditions were achieved (Wallin et al., 2021; Grandjean et al., 2023).

T2-weighted anatomical images were acquired at the start of each session using a TurboRARE pulse sequence [8 averages; 35 slices; slice thickness, 400 μm; field of view (FOV), 38.4 × 38.4 mm; matrix size, 192 × 192; in-plane resolution, 200 × 200 μm; repetition time (TR), 1,500 ms; echo time (TE), 15 ms; Hennig et al., 1986]. Resting-state fMRI data were collected using a gradient-echo echoplanar imaging (EPI) sequence with optimized parameters (Gilbert et al., 2019): 4 runs of 400 volumes each; TR, 1,500 ms; TE, 15 ms; FOV, 38.4 × 38.4 mm; matrix size, 96 × 96; 35 slices; isotropic resolution, 400 μm; bandwidth, 280 kHz. Image processing was performed using the Rodent Automated Bold Improvement of EPI Sequences (RABIES) software in conjunction with the SIGMA rat brain template (Barrière et al., 2019; Desrosiers-Grégoire et al., 2024). A detailed description of the workflow used for fMRI data processing was described in a previous study (Grandjean et al., 2023; Frie et al., 2024). Labeled images in native space produced during the RABIES preprocessing stage were used to extract region-specific masks. Raw time series from each mask were averaged across all voxels within the region and plotted. Each time series was normalized to a 0–1 range.

## Results

**Figure 5.**
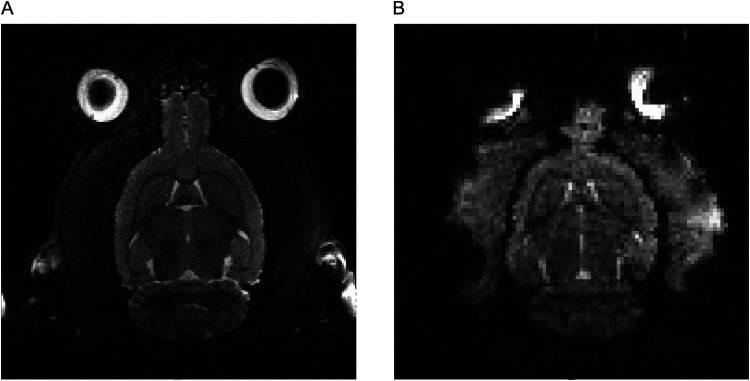
MRI images acquired using the rat restraint system. ***A***, T2-weighted anatomical image. ***B***, Functional MRI image from the first volume of the first scanning run in an awake, head-fixed rat.

Following the design and 3D printing of the rat restraint system, rats were habituated to restraint and head-fixing over a period of 11 d. After habituation, rats were placed in the MRI scanner for imaging. Each session began with T2-weighted image acquisition, followed by four fMRI runs. The full scanning session lasted up to 2 h, during which the rat remained awake and motion restricted. Representative T2-weighted and functional MRI images from an awake rat are shown in Figure 5*A*,*B*, respectively.

The T2-weighted axial image demonstrates high anatomical contrast and clear delineation of major brain structures, including the neocortex, cerebellum, and ventricular spaces, with minimal evidence of motion artifacts. The corresponding fMRI slice shows adequate signal homogeneity and preservation of anatomical landmarks despite lower spatial resolution, supporting reliable coregistration with the structural image. Minor susceptibility-related artifacts are visible near the eyes and olfactory bulbs, as expected with echoplanar imaging, but no significant motion-related distortions are observed.

**Figure 6.**
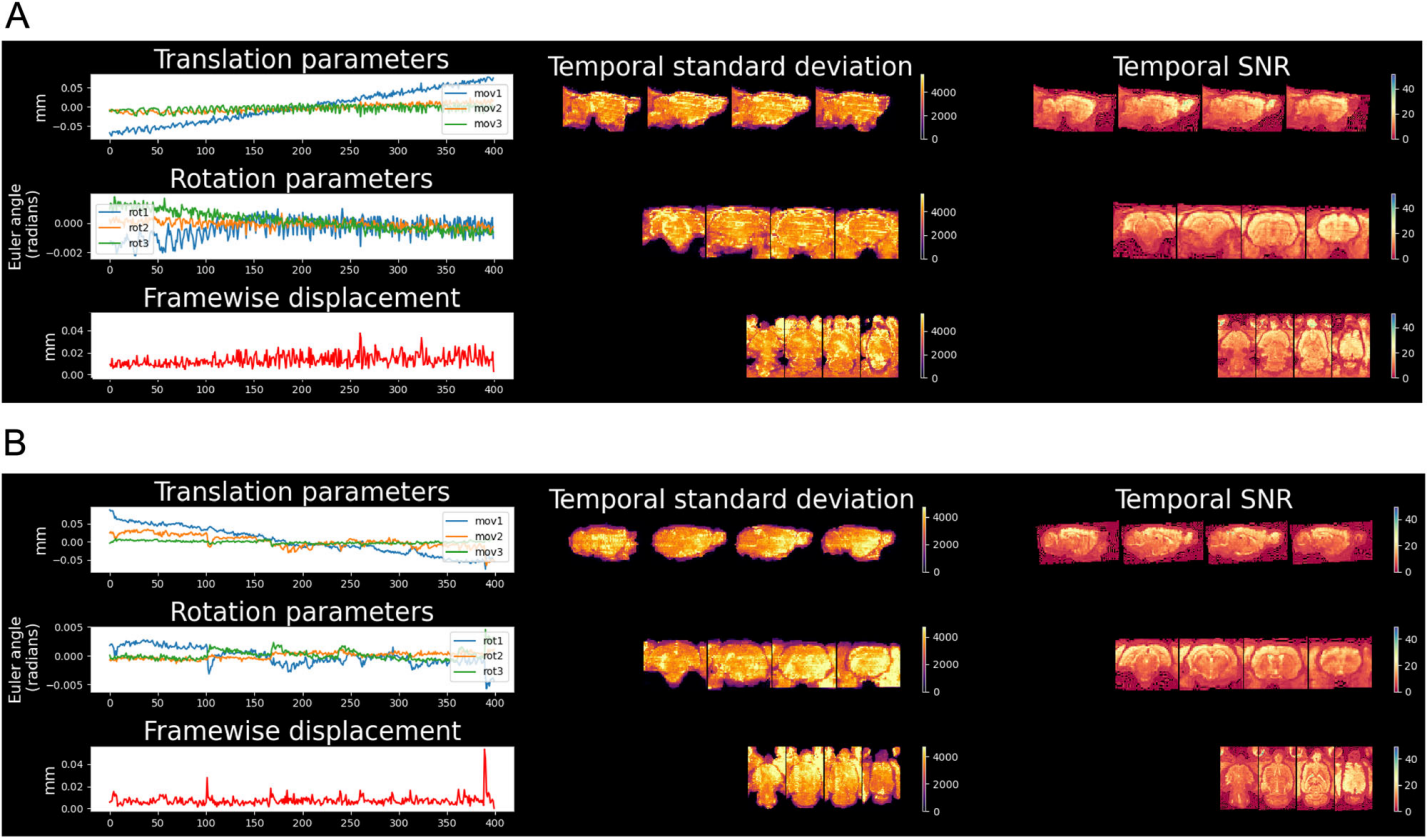
Representative average raw time series from multiple regions in anesthetized and awake rats. Left, Masks of the corresponding regions overlaid on the SIGMA template anatomical image. Middle, Average raw time series from an anesthetized rat. Right, Average raw time series from an awake rat. CA1v, ventral Cornu Ammonis 1; R-Par, right auditory parietal cortex; L-S1, left primary somatosensory cortex; R-S1, right primary somatosensory cortex; Hyp1, hypothalamus 1; Ins2, insular cortex 2; Cg2, cingulate cortex 2; PrL, prelimbic cortex; RSC1, retrosplenial cortex; M1/M2, primary and secondary motor cortex; L-dHip, left dorsal hippocampus; Str, striatum.

Representative raw BOLD time series were extracted from regions used in the functional connectivity analysis to qualitatively assess signal quality (Fig. 6). Shown are normalized BOLD signals from 12 functional brain regions (CA1v, R-Par, L-S1, R-S1, Hyp1, Ins2, Cg2, PrL, RSC1, M1/M2, L-dHip, and Str), obtained by averaging voxel-wise signals within each region's mask in both anesthetized and awake rats. Across all regions, the time series exhibited stable fluctuations throughout the scan duration, with no evidence of signal dropout, saturation, or prolonged drift. Following normalization, signal amplitudes remain within a consistent dynamic range, and fluctuations occur on timescales characteristic of low-frequency BOLD activity typically analyzed in resting-state fMRI. Together, these features support preservation of BOLD temporal structure and adequate signal stability, supporting the suitability of the data for subsequent functional connectivity analyses.

**Figure 7.**
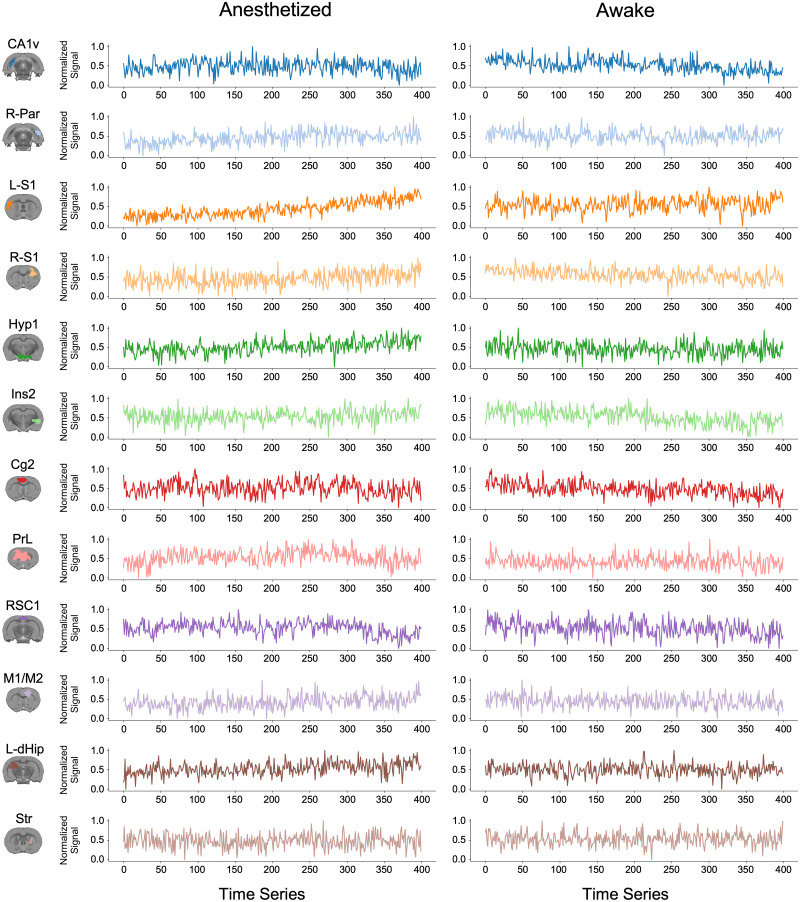
Head motion and image quality metrics during fMRI scanning. ***A***, Head motion and image quality metrics in an anesthetized rat. ***B***, Head motion and image quality metrics in an awake rat. Left panel, Top, Time course of translational head motion parameters; Middle, Rotational motion parameters; and Bottom, Framewise displacement across the scanning session. Middle panel, Temporal standard deviation maps displayed in sagittal (top), coronal (middle), and axial (bottom) views. Right panel, Temporal signal-to-noise ratio maps for sagittal (top), coronal (middle), and axial (bottom) views. Slice locations are reported in millimeters relative to bregma. Sagittal slices (mediolateral), 4.6, 3.4, 1.9, and 0.4 mm; coronal slices (anteroposterior), −7.30, −6.04, −1.30, and 2.70 mm; transverse slices (dorsoventral), −7.10, −6.10, −4.28, and −3.38 mm. Temporal standard deviation maps represent voxel-wise signal variability (arbitrary signal units), with higher values indicating lower temporal variability and lower values indicating higher temporal variability. Temporal signal-to-noise ratio (tSNR) maps are unitless and represent the ratio of the mean signal to its temporal standard deviation, with higher values indicating greater signal stability over time.

Head motion and signal quality metrics were assessed using the RABIES preprocessing pipeline. As shown in Figure 7*A*,*B* (head motion and signal quality metrics for a representative anesthetized and awake rat, respectively), both subjects exhibited minimal head motion throughout the scan. Translational head motion along the *x*- and *y*-axes were significantly lower, but significantly higher along the *z*-axis, in anesthetized rats compared with awake rats (all *p* < 0.0001, *n* = 5/group), while rotational displacements along all three axes were significantly lower in anesthetized rats compared with awake rats (all *p* < 0.0001), as shown in Table 1. Framewise displacement values were also significantly lower in anesthetized rats compared with awake rats (*p* < 0.0001). Temporal standard deviation maps revealed the expected spatial distribution of signal variability, with higher values localized to cortical and subcortical regions. These areas typically exhibit greater BOLD fluctuations due to physiological activity. Temporal signal-to-noise ratio maps demonstrated robust signal quality across the brain, with values exceeding 30 in most regions. These results confirm that the data quality is sufficient for subsequent analyses, with minimal noise contamination and adequate BOLD sensitivity across the scanned volume.

**Table 1.** Comparison of head motion parameters, including translation and rotation along the *x*-, *y*-, and *z*-axes, as well as framewise displacement between anesthetized and awake rats

Movement type	Direction	Awake (*n* = 5)	Anesthetized (*n* = 5)	*p* value
Translation (mm)	*x*	0.0333 ± 0.0213	0.0247 ± 0.0180	<0.0001
	*y*	0.0206 ± 0.0102	0.0082 ± 0.0051	<0.0001
	*z*	0.0059 ± 0.0035	0.0099 ± 0.0058	<0.0001
Rotation (rads)	*x*	0.0013 ± 0.0007	0.0008 ± 0.0004	<0.0001
	*y*	0.0004 ± 0.0002	0.0002 ± 0.0001	<0.0001
	*z*	0.0012 ± 0.0007	0.0003 ± 0.0002	<0.0001
Framewise Displacement (mm)	–	0.0114 ± 0.0057	0.0082 ± 0.0025	<0.0001

*x*, along the *x*-axis; *y*, along the *y*-axis; and *z*, along the *z*-axis. Sig., significant difference at *p* < 0.05.

**Figure 8.**
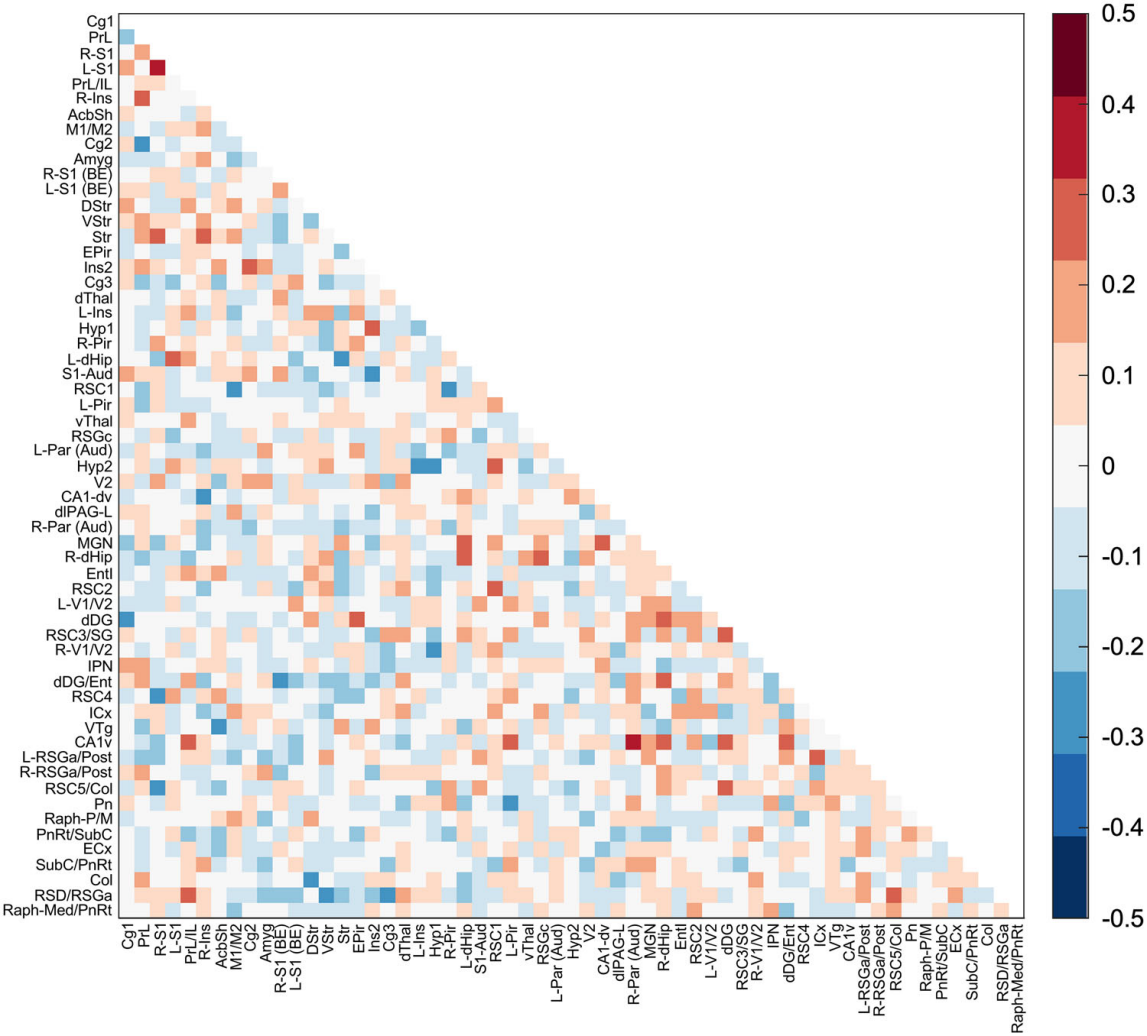
Average *Z*-scored functional connectivity matrix showing the difference (awake–anesthetized) in pairwise functional connectivity between awake and anesthetized rats. The matrix shows pairwise *Z*-transformed Pearson’s correlation coefficients between BOLD signals across 59 brain regions. Warmer colors indicate stronger positive connectivity in awake rats, and cooler colors indicate weaker or negative connectivity in awake rats compared with anesthetized rats.

We compared *Z*-scored functional connectivity (FC) matrices derived from resting-state fMRI data in awake and anesthetized rats (Fig. 8). This analysis revealed widespread FC differences between groups, with some region pairs showing increased and others showing decreased connectivity. Among the increases observed in awake rats relative to anesthetized rats, the largest effects were seen between the left and right primary somatosensory cortex (L-S1–R-S1; *z*-score: ≈+0.4), ventral Cornu Ammonis 1 and right auditory parietal cortex [CA1v–R-Par (Aud); *z*-score: ≈+0.4], and the hypothalamus and insula (Hyp1–Ins2; *z*-score: ≈+0.3). In contrast, the largest decreases in FC in awake rats were observed between the cingulate and prelimbic cortices (Cg2–PrL; *z*-score: ≈−0.3), retrosplenial and motor cortices (RSC1–M1/M2; *z*-score: ≈−0.3), and the right visual cortex and hypothalamus (R-V1/V2–Hyp1; *z*-score: ≈ − 0.3). However, none of these differences survived correction using NBS.

## Discussion

Our rat restraint system was designed to greatly minimize head motion and ensure animal comfort, without induction anesthesia use, thereby enabling the acquisition of high-quality images. The entire system was 3D printed over the course of 1 week, is easy to assemble, and can be modified to support additional behavioral tasks during scanning. The habituation protocol is straightforward and reliably acclimates rats to awake scanning within 11 d. Generally, head motion was higher in awake rats than in anesthetized rats. Moreover, functional connectivity in awake rats was stronger between some region pairs and weaker between others compared with anesthetized rats.

Prior to restraint system use, surgery is required to implant a head post. Although invasive, this approach remains the gold standard for studies requiring the highest spatial and temporal resolution. Noninvasive systems may be preferred as they eliminate the need for surgery (Stenroos et al., 2018; Ferris, 2022); however, they typically lack the mechanical rigidity needed to prevent submillimeter head motion, particularly during extended scans or behavioral task engagement. In contrast, head post-based systems provide superior head immobilization, facilitating the acquisition of motion-free images across long sessions and enabling precise image registration across time points (King et al., 2005; Tsurugizawa et al., 2020). Despite the surgical requirements, invasive systems remain the most reliable option when data fidelity, spatial accuracy, and compatibility with multimodal techniques are essential. The performance of our system demonstrates this advantage, yielding high-resolution T2-weighted and functional images with minimal motion artifacts.

Representative raw BOLD time series from multiple brain regions further support the quality of the fMRI data acquired with this awake imaging setup. The signals show stable low-frequency fluctuations across the entire scan, with no abrupt discontinuities, sustained baseline shifts, or large-amplitude spikes indicative of scanner artifacts, physiological instability, or excessive motion. This stability suggests that the head-fixation and habituation procedures were sufficient to preserve signal integrity during awake scanning. Although awake imaging in our study involved greater motion than anesthesia, these factors did not dominate or obscure the neural signals of interest.

Overall, framewise displacement, translation, and rotation were significantly higher in awake rats than in anesthetized rats during scanning, with the exception of translation along the *z*-axis, which was lower in awake animals. This pattern is expected, as anesthetized rats are largely immobilized. The reduced *z*-axis translation in awake rats likely reflects the head-fixation setup, which restricts vertical movement, whereas anesthetized rats can exhibit small *z*-axis shifts due to breathing. Importantly, although motion was greater in awake rats than in anesthetized rats, the magnitude of movements observed with our restraint system was lower than that reported in previous studies using other restraint systems (Stenroos et al., 2018). This highlights the effectiveness of our design in minimizing motion-related artifacts and improving fMRI signal quality in awake rats.

*Z*-scored FC comparisons revealed widespread differences in regions that form part of the rat default mode network (DMN; Lu et al., 2012). Specifically, awake rats showed higher connectivity between hippocampal and hypothalamic regions and lower connectivity involving the retrosplenial, prefrontal, and visual cortices compared with anesthetized rats. These patterns are consistent with prior studies demonstrating anesthesia-related alterations in similar DMN regions, particularly when contrasting awake and isoflurane conditions (Paasonen et al., 2018; Hori et al., 2020; Tsurugizawa and Yoshimaru, 2021; Gutierrez-Barragan et al., 2022). Collectively, these findings reinforce evidence that resting-state FC differs markedly between awake and anesthetized conditions and that such effects depend on both anesthetic type and dose (Liu et al., 2013; Jonckers et al., 2014). These anesthesia-dependent variations might confound findings from preclinical fMRI studies. The proposed restraint system and protocol enable awake imaging without anesthesia, helping preserve native FC patterns and improve data quality and replicability.

The development of effective restraint systems is central to advancing awake rodent fMRI, given the need to balance high-quality imaging with minimal stress and maximal animal welfare. Our system addresses several limitations inherent in existing designs by eliminating anesthesia use, reducing animal discomfort, and enabling behavioral integration during imaging. The avoidance of isoflurane is particularly critical, as even low doses altered functional connectivity in our anesthetized rats, potentially confounding results in studies aimed at capturing brain dynamics in naturalistic, awake conditions due to the remaining effects of induction anesthesia (Sloan, 1998; Martin et al., 2006; Paasonen et al., 2018). Instead, our approach supports scanning under fully conscious conditions, enabled by a restraint design that reduces head and body motion during imaging sessions.

Unlike traditional systems that rely on ear bars and bite bars for head immobilization, our design eliminates these features entirely. This modification significantly reduces discomfort, a known source of stress and motion in head-fixed preparations. The system's modularity and 3D-printed components allow for scalable adaptation, such as interchangeable tube inserts that accommodate rats of various sizes, sex, and ages, facilitating longitudinal studies and developmental imaging protocols. Another key advantage is the system's potential compatibility with behavioral paradigms. Because most of the rat's face, including the eyes, remains accessible during restraint, additional behaviors such as pupil tracking, requiring only an MRI-compatible camera positioned near the head, and lick-based tasks, using a small MRI-compatible lickometer (Frie and Khokhar, 2024) for liquid reward delivery or cue-driven licking, can be integrated during scanning. Although some locomotor behaviors requiring substantial limb movement, such as treadmill locomotion, are constrained by the current bore diameter, we are developing an expanded version of the system that will incorporate a low-profile roller-based treadmill, similar to that used in a previous study (Vasilev et al., 2022). This enables simultaneous acquisition of brain-wide activity and behavior in awake, task-engaged animals (Lawen et al., 2025), and integration with other open-source tools like fiber optic lickometers (Silva et al., 2024), or ability to measure the same consummatory behaviors in home-cage and in-magnet (Frie and Khokhar, 2024). The streamlined fixation process also contributes to experimental efficiency, typically requiring only ∼5 min to prepare and position each rat for scanning.

Despite these strengths, the restraint system is not without limitations. Most notably, it restricts direct access to the skull, thereby limiting compatibility with techniques such as electrophysiology, optogenetics, or fiber photometry that require chronic implants or optical access. This limitation could be addressed by stereotaxically implanting MRI-compatible electrodes or fibers before cementing the head post, as demonstrated previously (Paasonen et al., 2022), by designing head posts that anchor around the skull periphery while leaving the central area accessible for multimodal recordings, by incorporating non-ferromagnetic inserts into the head post design (Quansah Amissah et al., 2023), or by implanting skull surface EEG electrode arrays under the head post (Ding et al., 2024). This study employed a custom-built RF coil, which was necessary to achieve compatibility with the head-fixation system. However, this may limit reproducibility and direct comparability with studies using other coil designs, a constraint common to many awake rodent fMRI implementations. Additionally, although the restraint minimizes gross head motion, fine-scale movements such as whisking and jaw motion can still introduce artifacts. While these were infrequent and manageable in our experience, further reduction may be possible through additional behavioral training. Acoustic noise during MRI scanning remains another concern, as it can be a stressor for awake animals. To mitigate this, we recommend noise attenuation strategies such as silicone earplugs. One of the primary sources of MRI-related acoustic noise is the rapid switching of magnetic field gradients (MacKinnon et al., 2025). A promising strategy to mitigate acoustic noise and enhance suitability for awake imaging is the use of zero echo time (ZTE)-based and related quiet pulse sequences, which employ near-constant gradient activity to substantially reduce acoustic noise (Paasonen et al., 2022; Dvořáková et al., 2024; Stenroos et al., 2024; Daley et al., 2026), minimizing stress-induced movement and signal distortions while improving image quality. Corticosterone levels, albeit confounded by blood draw procedures, were not measured during habituation and scanning, which limits our ability to objectively quantify stress across the protocol. Although reductions in agitation-related behaviors and consistent reward consumption were used as indirect indicators of reduced stress, future studies should incorporate serial corticosterone measurements to provide an objective assessment of stress burden. Another potential confound is the inclusion of two rat strains, which on the one hand shows broader applicability of this design but also introduces potential confounds. Long–Evans and Sprague Dawley rats are known to differ in behavior and stress responsiveness (Schwarz et al., 2010). Although we observed no clear strain-related differences during restraint or scanning, the small sample size limits detection of subtle effects. Future studies with strain-balanced cohorts are needed to evaluate strain-dependent differences in habituation and motion during awake fMRI.

Our restraint system represents a significant step toward behaviorally compatible and reproducible awake rodent fMRI, especially given the lack of anesthesia use. By improving comfort, reducing motion, and supporting a range of behavioral tasks, it enables studies that closely approximate natural brain states. Future iterations may seek to enhance access for multimodal applications (e.g., electrophysiology; optical imaging) and further mitigate the impact of residual motion and acoustic stressors.

## Synthesis

Reviewing Editor: Katrina Choe, McMaster University Decisions are customarily a result of the Reviewing Editor and the peer reviewers coming together and discussing their recommendations until a consensus is reached. When revisions are invited, a fact-based synthesis statement explaining their decision and outlining what is needed to prepare a revision will be listed below. The following reviewer(s) agreed to reveal their identity: Sheng Song, Petteri Stenroos.

Both reviewers were in agreement that this work is a valuable contribution to the field of awake rodent fMRI. Reviewers also commented positively on the practicality and utility of this head restraint method for eliminating anesthesia, especially for longitudinal studies. They had several suggestions for the manuscript, listed below, which would further improve clarity and reproducibility.

Reviewer 1

Major Comments

1. The habituation protocol is currently provided only in narrative form, which makes it difficult to follow step-by-step. Since the training schedule is critical for achieving stable awake imaging, I recommend adding a simple visual workflow diagram summarizing the day-by-day progression (e.g., exploration → body restraint → head fixation → mock scanner exposure) along with session durations. A timeline figure would make the protocol considerably easier for others to implement accurately.

2. Because the primary aim of the system is to enable stable awake scanning, it would be helpful to include a brief performance comparison with scans acquired under anesthesia using the same hardware and acquisition parameters. This does not require additional functional interpretation. A small dataset (e.g., n = 2-3) demonstrating differences in mean framewise displacement and susceptibility-related distortion / anatomical alignment would clearly show the practical advantage of the awake preparation. This comparison would strengthen the methodological contribution without expanding the scope of the study.

3. Motion traces are currently shown for a single animal. Providing a simple summary table with mean {plus minus} SD of motion metrics (e.g., FD, translation, rotation) across animals would help define the typical performance range of the system and improve reproducibility for labs adopting the method.

4. The manuscript describes the habituation schedule, but it does not specify how the authors determined when an animal was considered fully habituated. For reproducibility, it would be helpful to state the behavioral criteria used to define habituation completion.

Additionally, since corticosterone is commonly used in the awake fMRI field to assess long-term stress burden, the authors may consider reporting corticosterone levels across the habituation period. This would further support the usability and translational relevance of the method.

Minor Comments

1. It would be helpful to clarify that the neck plate rests at the shoulder girdle / thoracic inlet, rather than on the cervical airway. A brief clarification in the text would reduce potential misinterpretation of restraint positioning.

2. If data are available, providing an approximate proportion of animals completing the full habituation duration would assist readers in planning timeline and throughput expectations.

3. The functional connectivity matrix could be made easier to interpret by adding region labels or modular grouping, although this is not essential to the core contribution.

Since the gradient insert bore is relatively small, it would be helpful for the authors to briefly comment on the scope for integrating concurrent behavioral tasks during awake scanning. In particular, clarifying which types of head-fixed compatible behaviors are feasible within the current spatial constraints would help readers understand the potential for future behavioral extensions of the setup.

Reviewer 2

Major:

1. Several times throughout the manuscript, the authors mention the reduced stress by the introduced protocol. Although, it might be true that stress levels are reduced by the fact that anesthesia was not used, it has not been proved by showing any physiological stress indicators or by referring to any previous article showing the correlation between stress and anesthesia use. Therefore, I would encourage the authors to either downplay the claim of reduced stress (by the fact of not using anesthesia) or show any measured physiological markers or add references to show the link between anesthesia and reduced stress.

2. Habitutation schedule of 11 days was used. Was it based on previous literature or observed motion level of rats?

3. Based on the correlation matrix, very weak resting state correlation is observed. The authors state this in the discussion, but in my opinion should be discussed further where it arises. Typically, resting state correlations between cortical regions are order of magnitude greater in awake animals than reported here (see DOIs: 10.1016/j.neuroimage.2021.118413 and 10.3389/fnins.2018.00548). It raises some concerns e.g. if the signal is influenced by some unwanted noise (system related or biological), which could be a reason for low correlation values. Which kind of filtering was used for the raw fMRI signal before generating correlation matrix? More details about the analysis should be added to method section. Could authors provide some raw fMRI signals from brain areas used for the analysis to be included for the response or potentially to the supplementary material?

4. The authors mention the method being suitable for behavioral studies using a treadmill (I assume with some modifications done first to the body tube). Have authors tried the rigidity of the head restraint alone without fully restricting the body or having a bigger body tube? With the neck support used is the position of rat in a natural position so that it can be potentially walking on top the treadmill comfortably. Some clarifications to these could help with the potential researchers thinking about the suitability of the system for their studies.

5. What was the sound pressure of the T2-weighted anatomical scans? Did the rats react to this sequence differently compared to GE-EPI sequence for example by moving?

6. For the males, Sprague Dawley and Long Evans strains were used, but any explanation for the reasons to use of two different strains is not given. Did the strains behave differently in the restraint setup? Please add some explanation or discuss further in the discussion about the potential behavioral differences.

Minor:

7. Please add to the methods, what was the reward used after habituation sessions.

8. In the introduction, the reliance of custom hardware is mentioned as a limitation for awake rodent fMRI. This is somewhat contradictory statement as custom RF-coil was used in the presented manuscript. The use of custom build RF-coil should be also added to the limitations chapter in the discussion. Also, as authors already mentioned, one other limitation of the restraint method is the use of anchoring system that is not suitable for multimodal imaging such as EEG/fMRI. Authors could refer to the already published manuscript (doi: 10.1016/j.neuroimage.2022.118924) that allows simultaneous EEG/fMRI measurement.

9. It should be noted that zero echo time sequence presented by MacKinnon et al., 2025 is not the original reference to show benefits of ZTE approach for awake imaging. The original works implementing zero echo time sequences as a quiet sequence for awake animal imaging, should be used as references (dois: 10.1016/j.neuroimage.2022.118924, 10.7554/eLife.90318, 10.1162/imag_a_00384), while work by MacKinnon can be mentioned as refinement of method using slightly different gradient waveform, resulting marginally smaller sound pressure than the original work using MB-SWIFT or ZTE approaches.

10. For the figure 5, please add bregma coordinates for the image slices presented for temporal standard deviation and temporal SNR. Also please add units/explanation for the color bar.

11. In lines 78 and 216, the sentences (with "XXX"-markings) should be corrected.

12. Typo in the line 161 (remove the "the rat to"?).

## Author Response

Dear Editor, We thank the reviewers and the editorial team for their constructive comments and feedback. Below, we have responded to the reviews provided by each referee, and we have correspondingly updated the manuscript for resubmission. We believe these changes have significantly improved the manuscript and we hope that it will be deemed suitable for publication in eNeuro. We will continue to build upon the findings from this manuscript, and look forward to incorporating the suggestions from the reviewers in future studies.

We confirm that neither the manuscript nor any parts of its content are currently under consideration or published in another journal. All authors have approved the manuscript and agree with its submission to eNeuro.

Sincerely, Jibran Khokhar Responses to Editor and Reviewers:

Thank you to the editor and reviewers for their continued insight and help with our manuscript. We have addressed the comments below.

Synthesis of Reviews:

Computational Neuroscience Model Code Accessibility Comments for Author (Required):

N/A Synthesis Statement for Author (Required):

Both reviewers were in agreement that this work is a valuable contribution to the field of awake rodent fMRI. Reviewers also commented positively on the practicality and utility of this head restraint method for eliminating anesthesia, especially for longitudinal studies. They had several suggestions for the manuscript, listed below, which would further improve clarity and reproducibility.

Reviewer 1 Major Comments 1. The habituation protocol is currently provided only in narrative form, which makes it difficult to follow step-by-step. Since the training schedule is critical for achieving stable awake imaging, I recommend adding a simple visual workflow diagram summarizing the day-by-day progression (e.g., exploration → body restraint → head fixation → mock scanner exposure) along with session durations. A timeline figure would make the protocol considerably easier for others to implement accurately.

Response We have added a visual workflow diagram to the "Steps for habituation to restraint, head fixing, and scanner noise" section as Figure 3, as suggested by the reviewer, seen below.

We have also referenced this figure throughout the manuscript on lines 164, 169, 171, 192, 205, and 220.

Figure 3. Visual flow diagram summarizing the step-by-step procedure for habituating a rat to the restraint system using the habituation protocol.

2. Because the primary aim of the system is to enable stable awake scanning, it would be helpful to include a brief performance comparison with scans acquired under anesthesia using the same hardware and acquisition parameters. This does not require additional functional interpretation. A small dataset (e.g., n = 2-3) demonstrating differences in mean framewise displacement and susceptibility-related distortion / anatomical alignment would clearly show the practical advantage of the awake preparation. This comparison would strengthen the methodological contribution without expanding the scope of the study.

Answer To compare performance between awake and anesthetized rats, we have included data on translation, rotation, and framewise displacement during scanning for both groups in Table 1, as shown below.

We have updated the text on head motion in the results section as follows on lines 302 to 309: "As shown in Fig. 7A and B (head motion and signal quality metrics for a representative anesthetized and awake rat, respectively), both subjects exhibited minimal head motion throughout the scan. Translational head motion along the x and y axes were significantly lower, but significantly higher along the z axis, in anesthetized rats compared to awake rats (all p < 0.0001, n = 5/group), while rotational displacements along all three axes were significantly lower in anesthetized rats compared to awake rats (all p < 0.0001), as shown in Table 1. Framewise displacement values were also significantly lower in anesthetized rats compared to awake rats (p < 0.0001)." Table 1: Comparison of head-motion parameters, including translation and rotation along the x, y, and z axes, as well as framewise displacement between anesthetized and awake rats.

We have also included additional text in the discussion section as follows on lines 357 to 366: "Overall, framewise displacement, translation, and rotation were significantly higher in awake rats than in anesthetized rats during scanning, with the exception of translation along the z-axis, which was lower in awake animals. This pattern is expected, as anesthetized rats are largely immobilized. The reduced z-axis translation in awake rats likely reflects the head-fixation setup, which restricts vertical movement, whereas anesthetized rats can exhibit small z-axis shifts due to breathing. Importantly, although motion was greater in awake rats than in anesthetized rats, the magnitude of movements observed with our restraint system was lower than that reported in previous studies using other restraint systems (Stenroos et al., 2018). This highlights the effectiveness of our design in minimizing motion-related artifacts and improving fMRI signal quality in awake rats." To extend the comparison between awake and anesthetized rats, we compared functional connectivity in awake and anesthetized rats as well. We have replaced the previous functional connectivity matrix heat map (Figure 8) with a new heat map representing the differences (changes) in functional connectivity between anesthetized and awake rats, as seen below.

Figure 8. Average Z-scored functional connectivity matrix showing the difference (awake - anesthetized) in pairwise functional connectivity between awake and anesthetized rats. The matrix shows pairwise Z-transformed Pearson correlation coefficients between BOLD signals across 59 brain regions. Warmer colors indicate stronger positive connectivity in awake rats, and cooler colors indicate weaker or negative connectivity in awake rats compared to anesthetized rats.

The results of this analysis have also been described in the results section on lines 316 to 326 as follows: "We compared Z-scored functional connectivity (FC) matrices derived from resting-state fMRI data in awake and anesthetized rats (Fig. 8). This analysis revealed widespread FC differences between groups, with some region pairs showing increased and others showing decreased connectivity. Among the increases observed in awake rats relative to anesthetized rats, the largest effects were seen between the left and right primary somatosensory cortex (L-S1-R-S1; z-score: l +0.4), ventral Cornu Ammonis 1 and right auditory parietal cortex (CA1v-R-Par (Aud); z-score: +0.4), and the hypothalamus and insula (Hyp1-Ins2; z-score: +0.3). In contrast, the largest decreases in FC in awake rats were observed between the cingulate and prelimbic cortices (Cg2-PrL; z-score: -0.3), retrosplenial and motor cortices (RSC1-M1/M2; z-score: / -0.3), and the right visual cortex and hypothalamus (R-V1/V2-Hyp1; z-score: / -0.3). However, none of these differences survived correction using NBS," and has been discussed in the discussion section as follows on lines 334 to 336: "Moreover, functional connectivity in awake rats was stronger between some region pairs and weaker between others compared with anesthetized rats," and on lines 367 to 379: "Z-scored FC comparisons revealed widespread differences in regions that form part of the rat default mode network (DMN) (Lu et al., 2012). Specifically, awake rats showed higher connectivity between hippocampal and hypothalamic regions and lower connectivity involving the retrosplenial, prefrontal, and visual cortices compared with anesthetized rats. These patterns are consistent with prior studies demonstrating anesthesia-related alterations in similar DMN regions, particularly when contrasting awake and isoflurane conditions (Paasonen et al., 2018; Hori et al., 2020; Tsurugizawa and Yoshimaru, 2021; Gutierrez-Barragan et al., 2022). Collectively, these findings reinforce evidence that resting-state FC differs markedly between awake and anesthetized conditions and that such effects depend on both anesthetic type and dose (Liu et al., 2013; Jonckers et al., 2014). These anesthesia-dependent variations might confound findings from preclinical fMRI studies. The proposed restraint system and protocol enable awake imaging without anesthesia, helping preserve native FC patterns and improve data quality and replicability." 3. Motion traces are currently shown for a single animal. Providing a simple summary table with mean {plus minus} SD of motion metrics (e.g., FD, translation, rotation) across animals would help define the typical performance range of the system and improve reproducibility for labs adopting the method.

Answer We have added a table (Table 1) summarizing the mean W SD of motion metrics (translation, rotation, and framewise displacement) for all awake and anesthetized rats, illustrating the typical performance range of the restraint system. Corresponding descriptions have been added to the Results section and further addressed in the Discussion, as detailed above.

We have also updated Figure 6 by including a second motion trace for a representative anesthetized rat for comparison.

Figure 7. Head motion and image quality metrics during fMRI scanning. A. Head motion and image quality metrics in an anesthetized rat. B. Head motion and image quality metrics in an awake rat. Left panel: Top: Time course of translational head motion parameters, Middle: Rotational motion parameters, and Bottom: Framewise displacement across the scanning session. Middle panel: Temporal standard deviation maps displayed in sagittal (top), coronal (middle), and axial (bottom) views. Right panel: Temporal signal-to-noise ratio maps for sagittal (top), coronal (middle), and axial (bottom) views.

We have also added a corresponding text to the Results section as follows on lines 302 to 309: "As shown in Fig. 7A and B (head motion and signal quality metrics for a representative anesthetized and awake rat, respectively), both subjects exhibited minimal head motion throughout the scan. Translational head motion along the x and y axes were significantly lower, but higher along the z axis, in anesthetized rats compared to awake rats (all p < 0.0001; n=5/group), while rotational displacements along all three axes were significantly lower in anesthetized rats compared to awake rats (all p < 0.0001), as shown in Table 1. Framewise displacement values were also significantly lower in anesthetized rats compared to awake rats (p < 0.0001)." 4. The manuscript describes the habituation schedule, but it does not specify how the authors determined when an animal was considered fully habituated. For reproducibility, it would be helpful to state the behavioral criteria used to define habituation completion.

Answer While these behaviors were not formally quantified, we relied on consistent qualitative indicators that the animals reached a stable, habituated state during restraint. By approximately the 11th day of habituation, all animals met the following criteria: they could be placed into the restraint and head-fixation apparatus without resistance; they displayed minimal or no tail flicks, body twisting, or escape attempts during immobilization; and they reliably consumed the food rewards provided at the end of each session. We have added the following text to the section "Steps for habituation to restraint, head fixing, and scanner noise" on lines 224 to 228: "By the final day of habituation, rats were introduced into the restraint and head-fixation setup without resistance, and exhibited minimal or no tail flicks, body twisting, or escape attempts. They also produced fewer fecal pellets during this session and consistently consumed the rewards offered after each session. These behaviors were used as qualitative criteria to determine completion of habituation." 5. Additionally, since corticosterone is commonly used in the awake fMRI field to assess long-term stress burden, the authors may consider reporting corticosterone levels across the habituation period. This would further support the usability and translational relevance of the method.

Answer We agree that corticosterone is a valuable biomarker for assessing long-term stress in awake fMRI paradigms. Unfortunately, we did not collect blood samples during this study and therefore cannot report corticosterone levels across the habituation period. Instead, we relied on qualitative behavioral indicators, such as minimal or no tail flicks, body twisting, or escape attempts, number of fecal pellets produced, and consistent consumption of rewards by the final days of habituation, as indirect signs of reduced stress. We acknowledge that these behavioral observations do not substitute for an objective biochemical measure, and we have added this point to the limitations section of the manuscript as follows on lines 433 to 438: "Corticosterone levels, albeit confounded by blood draw procedures, were not measured during habituation and scanning, which limits our ability to objectively quantify stress across the protocol. Although reductions in agitation-related behaviors and consistent reward consumption were used as indirect indicators of reduced stress, future studies should incorporate serial corticosterone measurements to provide an objective assessment of stress burden." Minor Comments 1. It would be helpful to clarify that the neck plate rests at the shoulder girdle / thoracic inlet, rather than on the cervical airway. A brief clarification in the text would reduce potential misinterpretation of restraint positioning.

Answer We thank the reviewer for this helpful suggestion. The neck plate does not rest on or apply pressure to the cervical airway. Instead, when lowered, it sits at the level of the thoracic inlet/shoulder girdle, where the shape of the plate and connector creates an adjustable circular opening that accommodates the rat's neck without constriction. This positioning ensures that the airway remains unobstructed and that the animals can breathe comfortably during immobilization and head fixation. We have added the following clarification to the "Steps for habituation to restraint, head fixing, and scanner noise" section on lines 183 to 187: "When lowered, the neck plate engages at the level of the shoulder girdle/thoracic inlet. The plate and connector form an adjustable circular opening that fits around the neck without compressing the cervical airway, ensuring unobstructed breathing during restraint." 2. If data are available, providing an approximate proportion of animals completing the full habituation duration would assist readers in planning timeline and throughput expectations.

Answer We thank the reviewer for this suggestion. In our dataset, 100% of the rats that began the habituation protocol (n = 5) successfully completed the full habituation schedule. Although animals show the greatest variability in resistance during the first immobilization and head-fixation sessions, all rats reliably adapted over repeated sessions, and by the final day no meaningful differences in compliance were observed. We have added the following clarification to the "Steps for habituation to restraint, head fixing, and scanner noise" section on lines 228 to 229: "Across all animals exposed to the protocol, 100% (n = 5) successfully completed the full habituation protocol." 3. The functional connectivity matrix could be made easier to interpret by adding region labels or modular grouping, although this is not essential to the core contribution.

Answer We have updated the figure for the functional connectivity matrix by making the region labels clearer. We have also referenced the atlas from which the definitions for these labels can be obtained in the section on "MRI Experiments" as follows on lines 267 to 270: "Image processing was performed using the Rodent Automated Bold Improvement of EPI Sequences (RABIES) software in conjunction with the SIGMA rat brain template (Barrière et al., 2019; Desrosiers-Grégoire et al., 2024)." Figure 8. Average Z-scored functional connectivity matrix showing the difference (awake - anesthetized) in pairwise functional connectivity between awake and anesthetized rats. The matrix shows pairwise Z-transformed Pearson correlation coefficients between BOLD signals across 59 brain regions. Warmer colors indicate stronger positive connectivity, and cooler colors indicate weaker or negative connectivity.

4. Since the gradient insert bore is relatively small, it would be helpful for the authors to briefly comment on the scope for integrating concurrent behavioral tasks during awake scanning. In particular, clarifying which types of head-fixed compatible behaviors are feasible within the current spatial constraints would help readers understand the potential for future behavioral extensions of the setup.

Answer We agree that the relatively small gradient insert bore limits the complexity and range of behavioral tasks that can be performed during awake scanning. We have modified the text to clarify the types of behaviors that are feasible within the existing spatial limitations on lines 395 to 403: "Another key advantage is the system's potential compatibility with behavioral paradigms. Because most of the rat's face, including the eyes, remains accessible during restraint, additional behaviors such as pupil tracking, requiring only an MRI-compatible camera positioned near the head, and lick-based tasks, using a small MRI-compatible lickometer (Frie and Khokhar, 2024) for liquid reward delivery or cue-driven licking, can be integrated during scanning. Although some locomotor behaviors requiring substantial limb movement, such as treadmill locomotion, are constrained by the current bore diameter, we are developing an expanded version of the system that will incorporate a low-profile roller-based treadmill, similar to that used in a previous study (Vasilev et al., 2022)." Reviewer 2 Major:

1. Several times throughout the manuscript, the authors mention the reduced stress by the introduced protocol. Although, it might be true that stress levels are reduced by the fact that anesthesia was not used, it has not been proved by showing any physiological stress indicators or by referring to any previous article showing the correlation between stress and anesthesia use. Therefore, I would encourage the authors to either downplay the claim of reduced stress (by the fact of not using anesthesia) or show any measured physiological markers or add references to show the link between anesthesia and reduced stress.

Answer We thank the reviewer for this important clarification. Because we did not collect physiological stress markers (e.g., corticosterone), we revised the manuscript to avoid making any unsubstantiated claims. Specifically, we removed language suggesting that the protocol "reduces stress" and instead restricted our statements to objectively observed behaviors during habituation (e.g., decreases in escape attempts and body movements). These revisions have been applied throughout the Significance Statement (lines 30 to 32: "By reducing motion, this simple restraint system allows for investigation of neural activity and connectivity without confounds from sedation or anesthesia."), Introduction (lines 69 to 71: "Our approach combines skull-screw-less head post implantation with a structured 11-day habituation regimen to minimize motion without the use of anesthesia."), and Methods (lines 164 to 165: "It is designed to minimize movement while preserving physiologically relevant brain states."), to ensure the text no longer implies that stress reduction was demonstrated.

2. Habituation schedule of 11 days was used. Was it based on previous literature or observed motion level of rats? Answer The 11-day habituation schedule was not based on prior literature but was determined from our observations during the habituation sessions. Although we did not formally quantify behavioral or physiological indicators, we noted progressive reductions in resistance to placement in the restraint system and minimal spontaneous movements (e.g., tail flicks, body twisting, escape attempts), as well as fewer fecal pellets by day 11, at which point animals appeared reliably acclimated. We agree that objective, quantifiable behavioral criteria would strengthen reproducibility. We have added the following text to the section "Steps for habituation to restraint, head fixing, and scanner noise" on lines 224 to 228: "By the final day of habituation, rats were introduced into the restraint and head-fixation setup without resistance, and exhibited minimal or no tail flicks, body twisting, or escape attempts. They also produced fewer fecal pellets during the restraint session and consistently consumed the rewards offered after each session. These behaviors were used as qualitative criteria to determine completion of habituation." 3. Based on the correlation matrix, very weak resting state correlation is observed. The authors state this in the discussion, but in my opinion should be discussed further where it arises. Typically, resting state correlations between cortical regions are order of magnitude greater in awake animals than reported here (see DOIs: 10.1016/j.neuroimage.2021.118413 and 10.3389/fnins.2018.00548). It raises some concerns e.g. if the signal is influenced by some unwanted noise (system related or biological), which could be a reason for low correlation values. Which kind of filtering was used for the raw fMRI signal before generating correlation matrix? More details about the analysis should be added to method section. Could authors provide some raw fMRI signals from brain areas used for the analysis to be included for the response or potentially to the supplementary material? Answer We thank the reviewer for highlighting this important issue. We agree that the initially reported resting-state correlations were substantially weaker (-0.03 to 0.03) than those typically observed in awake rodents and warranted closer examination. Upon re-evaluating our analysis pipeline, we identified that the functional connectivity matrices were not z-score normalized as intended due to an error in the analysis script. As a result, the values originally reported reflected unnormalized correlation coefficients. After correcting this issue, and regenerating the connectivity matrices using Fisher z-transformation, the magnitude of the difference between awake and anesthetized correlations was between -0.5 to 0.5; this is now comparable to values reported in prior awake rodent fMRI studies, including those cited by the reviewer, as seen below.

Figure 8. Average Z-scored functional connectivity matrix showing the difference in pairwise functional connectivity between awake and anesthetized rats. The matrix shows pairwise Z-transformed Pearson correlation coefficients between BOLD signals across 59 brain regions. Warmer colors indicate stronger positive connectivity, and cooler colors indicate weaker or negative connectivity.

To address the reviewer's question regarding preprocessing, the raw fMRI time series were processed using the RABIES pipeline, including motion correction, distortion correction, nuisance regression (motion parameters, white matter, CSF signals), global signal regression, and band-pass filtering (0.01-0.1 Hz; Grandjean et al., 2023; Desrosiers-Grégoire et al., 2024). These details can be found in the supplementary materials section of our recent paper (Frie et al, 2024).

We have also included representative raw fMRI signals from the brain regions used in the analysis as Fig. 6 in the main manuscript, as requested by the reviewer. We also added the following text to the Methods section on lines 271 to 274: "Labeled images in native space produced during the RABIES preprocessing stage were used to extract region-specific masks. Raw time series from each mask were averaged across all voxels within the region and plotted. Each time series was normalized to a 0-1 range." In addition, we added the following text to the Results section on lines 290 to 300: "Representative raw BOLD time series were extracted from regions used in the functional connectivity analysis to qualitatively assess signal quality (Fig. 6). Shown are normalized BOLD signals from 12 functional brain regions (CA1v, R-Par, L-S1, R-S1, Hyp1, Ins2, Cg2, PrL, RSC1, M1/M2, L-dHip, and Str), obtained by averaging voxel-wise signals within each region's mask in both anesthetized and awake rats. Across all regions, the time-series exhibited stable fluctuations throughout the scan duration, with no evidence of signal dropout, saturation, or prolonged drift. Following normalization, signal amplitudes remain within a consistent dynamic range, and fluctuations occur on timescales characteristic of low-frequency BOLD activity typically analyzed in resting-state fMRI. Together, these features support preservation of BOLD temporal structure and adequate signal stability, supporting the suitability of the data for subsequent functional connectivity analyses." We also added the following text to the Discussion section on lines 349 to 356: "Representative raw BOLD time series from multiple brain regions further support the quality of the fMRI data acquired with this awake imaging setup. The signals show stable low-frequency fluctuations across the entire scan, with no abrupt discontinuities, sustained baseline shifts, or large-amplitude spikes indicative of scanner artifacts, physiological instability, or excessive motion. This stability suggests that the head-fixation and habituation procedures were sufficient to preserve signal integrity during awake scanning. Although awake imaging in our study involved greater motion and physiological variability than anesthesia, these factors did not dominate or obscure the neural signals of interest." Figure 6. Representative average raw time series from multiple regions in anesthetized and awake rats. Left: masks of the corresponding regions overlaid on the SIGMA template anatomical image. Middle: average raw time series from an anesthetized rat. Right: average raw time series from an awake rat. CA1v, ventral Cornu Ammonis 1; R-Par, right auditory parietal cortex; L-S1, left primary somatosensory cortex; R-S1, right primary somatosensory cortex; Hyp1, hypothalamus 1; Ins2, insular cortex 2; Cg2, cingulate cortex 2; PrL, prelimbic cortex; RSC1, retrosplenial cortex; M1/M2, primary and secondary motor cortex; L-dHip, left dorsal hippocampus; Str, striatum.

4. The authors mention the method being suitable for behavioral studies using a treadmill (I assume with some modifications done first to the body tube). Have authors tried the rigidity of the head restraint alone without fully restricting the body or having a bigger body tube? With the neck support used is the position of rat in a natural position so that it can be potentially walking on top the treadmill comfortably. Some clarifications to these could help with the potential researchers thinking about the suitability of the system for their studies.

Answer We evaluated the rigidity of the head fixation independently of full-body restraint by testing a configuration in which only the head was fixed while the rat was positioned on a treadmill. In these tests, the head-post provided sufficient rigidity and stability for head fixation alone. However, in the current design, the neck-plate geometry positions the head and neck lower than would be required for a natural posture, which prevents comfortable or sustained walking. As noted by the reviewer, the restricted bore diameter and body-tube dimensions further limit limb movement. Consequently, while head fixation alone is mechanically stable, as shown below, the present configuration is not yet suitable for treadmill-based behavioral tasks. We are actively developing modified versions of the restraint system that incorporate a larger body tube, elevated head and neck support to better align the animal in a natural locomotor posture, and roller grid-based low-profile treadmills. These modifications are intended to enable proper stepping mechanics and facilitate future integration of treadmill walking during awake fMRI.

We have updated the text in the Discussion section as follows on lines 396 to 403: "Because most of the rat's face, including the eyes, remains accessible during restraint, additional behaviors such as pupil tracking, requiring only an MRI-compatible camera positioned near the head and lick-based tasks, using a small MRI-compatible lickometer (Frie and Khokhar, 2024) for liquid reward delivery or cue-driven licking, can be integrated during scanning. Although behaviors requiring substantial limb movement, such as treadmill locomotion, are constrained by the current bore diameter, we are developing an expanded version of the system that will incorporate a low-profile roller-based linear treadmill, similar to that used in a previous study (Vasilev et al., 2022)." 5. What was the sound pressure of the T2-weighted anatomical scans? Did the rats react to this sequence differently compared to GE-EPI sequence for example by moving? Answer The peak acoustic output of the MRI system used in this study is approximately 120 dB, and this applies to both the T2-weighted anatomical and GE-EPI functional sequences. During habituation, rats were repeatedly exposed to recorded scanner noise of comparable intensity to acclimate them to both sequence types. Although animals were not quantitatively monitored during mock scanner sessions, they were continuously observed during actual MRI acquisition. We did not observe any overt behavioral differences or increases in motion during the T2-weighted anatomical scans compared to the GE-EPI sequence. In particular, no sequence-specific increases in head movement, struggling, or agitation were apparent during anatomical imaging. While we acknowledge that these observations were qualitative and not quantitative, they suggest that the T2-weighted sequence did not elicit a stronger behavioral or motion response than the GE-EPI sequence in habituated animals and vice versa. This information is provided in the section "Steps for habituation to restraint, head fixing, and scanner noise" on lines 206 to 218 as follows: "After immobilization and head fixation, place the rat with the restraint system inside the mock MRI chamber and expose it to recorded MRI (T2-weighted anatomical and GE-EPI acquisition) sounds. i. Using a speaker capable of producing sounds up to 120 dB sound pressure level (SPL; the peak intensity of sounds produced by the MRI system used during both T2-weighted anatomical and GE-EPI sequences), play the recorded MRI sounds-starting at a lower intensity on day 7 and gradually increasing the intensity each day, reaching full intensity by day 11. ii. From days 7 to 11, immobilize the rat with head-fixing in the mock MRI chamber for progressively longer durations: 30, 45, 60, 90, and 120 minutes, respectively. No overt behavioral differences, such as head movement, struggling, or agitation, were observed during exposure to MRI sounds generated during either T2-weighted anatomical scans or GE-EPI sequence." 6. For the males, Sprague Dawley and Long Evans strains were used, but any explanation for the reasons to use of two different strains is not given. Did the strains behave differently in the restraint setup? Please add some explanation or discuss further in the discussion about the potential behavioral differences.

Answer Two male rat strains (Sprague Dawley and Long Evans) were included to test the broader applicability of the design across multiple rat strains. We acknowledge that the inclusion of multiple strains is a potential confound for strain-specific behavioral or stress-related differences during habituation and restraint. We have now added text to address this limitation to the Discussion section as follows on lines 438 to 444: "Another potential confound is the inclusion of two rat strains, which on one hand shows broader applicability of this design, but also introduces potential confounds. Long Evans and Sprague Dawley rats are known to differ in behavior and stress responsiveness (Schwarz et al., 2010). Although we observed no clear strain-related differences during restraint or scanning, the small sample size limits detection of subtle effects. Future studies with strain-balanced cohorts are needed to evaluate strain-dependent differences in habituation and motion during awake fMRI." Minor:

7. Please add to the methods, what was the reward used after habituation sessions.

Answer Banana-flavored sugar pellets were used as rewards after the habituation sessions (Bio-Serv, 45 mg, banana flavor; F0024). This information has been added to the Methods section as follows on lines 87 to 88: "Rats received banana-flavored sugar pellets (Bio-Serv, 45 mg, banana flavor, NJ, USA) as a reward after each habituation session." 8. In the introduction, the reliance of custom hardware is mentioned as a limitation for awake rodent fMRI. This is somewhat contradictory statement as custom RF-coil was used in the presented manuscript. The use of custom build RF-coil should be also added to the limitations chapter in the discussion. Also, as authors already mentioned, one other limitation of the restraint method is the use of anchoring system that is not suitable for multimodal imaging such as EEG/fMRI. Authors could refer to the already published manuscript (doi: 10.1016/j.neuroimage.2022.118924) that allows simultaneous EEG/fMRI measurement.

Answer We agree with the reviewer and have revised the manuscript accordingly. To address the apparent contradiction in the Introduction, we clarified that reliance on custom hardware is a general limitation of awake rodent fMRI rather than a flaw specific to our implementation. The revised text in the Introduction now reads on lines 56 to 59: "Despite its advantages, widespread adoption of awake rodent fMRI has been hindered by technical barriers, including procedural complexity, lack of standardized protocols, and reliance on custom hardware, which is often required to achieve adequate signal quality and head stabilization." We explicitly added the use of a custom-built RF coil to the Limitations section of the Discussion, as follows on lines 418 to 421: "This study employed a custom-built RF coil, which was necessary to achieve compatibility with the head-fixation system. However, this may limit reproducibility and direct comparability with studies using other coil designs, a constraint common to many awake rodent fMRI implementations." We also expanded the discussion of the system's limited compatibility with multimodal studies as follows on lines 413 to 418: "This limitation could be addressed by stereotaxically implanting MRI-compatible electrodes or fibers before cementing the head post, as demonstrated previously (Paasonen et al., 2022), by designing head posts that anchor around the skull periphery while leaving the central area accessible for multimodal recordings, by incorporating non-ferromagnetic inserts into the head post design (Quansah Amissah et al., 2023), or by implanting skull surface EEG electrode arrays under the head-post (Ding et al., 2024)." 9. It should be noted that zero echo time sequence presented by MacKinnon et al., 2025 is not the original reference to show benefits of ZTE approach for awake imaging. The original works implementing zero echo time sequences as a quiet sequence for awake animal imaging, should be used as references (dois: 10.1016/j.neuroimage.2022.118924, 10.7554/eLife.90318, 10.1162/imag_a_00384), while work by MacKinnon can be mentioned as refinement of method using slightly different gradient waveform, resulting marginally smaller sound pressure than the original work using MB-SWIFT or ZTE approaches.

Answer We have updated the Discussion section and cited the reviewer-recommended references as follows on lines 428 to 433: "A promising strategy to mitigate acoustic noise and enhance suitability for awake imaging is the use of zero echo time (ZTE)-based and related quiet pulse sequences, which employ near-constant gradient activity to substantially reduce acoustic noise (Paasonen et al., 2022; Dvořáková et al., 2024; Stenroos et al., 2024), minimizing stress-induced movement and signal distortions while improving image quality." 10. For the figure 5, please add bregma coordinates for the image slices presented for temporal standard deviation and temporal SNR. Also please add units/explanation for the color bar.

Answer We have revised the legend for Figure 7 to include both bregma coordinates and clear explanations of the color bars, including units. Specifically, we added the following text to the figure legend on lines 644 to 651: "Slice locations are reported in millimeters relative to bregma. Sagittal slices (mediolateral): 4.6, 3.4, 1.9, and 0.4 mm; coronal slices (anteroposterior): −7.30, −6.04, −1.30, and 2.70 mm; transverse slices (dorsoventral): −7.10, −6.10, −4.28, and −3.38 mm. Temporal standard deviation maps represent voxel-wise signal variability (arbitrary signal units), with higher values indicating lower temporal variability and lower values indicating higher temporal variability. Temporal signal-to-noise ratio (tSNR) maps are unitless and represent the ratio of the mean signal to its temporal standard deviation, with higher values indicating greater signal stability over time." 11. In lines 78 and 216, the sentences (with "XXX"-markings) should be corrected.

Response We used "XXX" as placeholders because the journal's double-blind review policy requires that no identifying information be included in the manuscript.

12. Typo in the line 161 (remove the "the rat to"?).

Answer We have modified the text as follows on lines 176 to 177: "Wrap the napkin around the rear of the tube and the rear of the rat to encourage it to move forward through." References Desrosiers-Grégoire G, Devenyi GA, Grandjean J, Chakravarty MM (2024) A standardized image processing and data quality platform for rodent fMRI. Nature Communications 15:6708 Grandjean J, Desrosiers-Gregoire G, Anckaerts C, Angeles-Valdez D, Ayad F, Barrière DA, Blockx I, Bortel A, Broadwater M, Cardoso BM (2023) A consensus protocol for functional connectivity analysis in the rat brain. Nature neuroscience 26:673-681.

Frie JA, McCunn P, Eed A, Hassan A, Luciani KR, Chen C, Tyndale RF, Khokhar JY (2024) Factors influencing JUUL e-cigarette nicotine vapour-induced reward, withdrawal, pharmacokinetics and brain connectivity in rats: sex matters. Neuropsychopharmacology 49:782-795 Schwarz, C., Hentschke, H., Butovas, S., Haiss, F., Stüttgen, M. C., Gerdjikov, T. V., ... &Waiblinger, C. (2010). The head-fixed behaving rat-procedures and pitfalls. Somatosensory &motor research, 27(4), 131-148.
